# Mapping immune trajectories from *Helicobacter pylori* gastritis to gastric cancer

**DOI:** 10.3389/fimmu.2025.1693004

**Published:** 2025-12-17

**Authors:** Yi Song, Jie Qiu, Jingyue Wu, Wenqi Liu, Qiang Wu

**Affiliations:** 1 Department of Gastroenterology, Suzhou Ninth People’s Hospital, Soochow University, Suzhou, Jiangsu, China; 2 Department of Gastrointestinal Surgery, Suzhou Ninth People’s Hospital, Soochow University, Suzhou, Jiangsu, China; 3 Hebei Medical University, Shijiazhuang, Hebei, China; 4 Department of Thoracic Surgery, Wuhan Hospital of Traditional Chinese And Western Medicine, Wuhan, China

**Keywords:** gastric cancer, *H. pylori*, immune exclusion, immune snapshot, intestinal metaplasia, risk stratification, therapy sequencing

## Abstract

*Helicobacter pylori*-associated gastric tumorigenesis is typically staged by histopathology, yet clinical decisions often hinge on immune dynamics that histology alone cannot resolve. This mini review introduces a phase-specific “immune snapshot” framework that integrates three elements—who is present in the microenvironment, how key circuits are engaged, and where these features reside—to track immune trajectories across chronic gastritis, atrophy with intestinal metaplasia, and dysplasia or early cancer. Rather than cataloging markers, we emphasized a practical approach that maps compact tissue and fluid assays to decision points, including when to normalize stroma and vasculature, when to prime antigenicity, and when to deploy checkpoint blockade. The novelty lies in linking stage-aware immune states to actionable sequencing of interventions and to risk-adapted surveillance intervals. By complementing histopathology with dynamic, mechanism-informed readouts that are feasible in routine practice, the framework aims to identify reversible barriers, clarify timing, and improve the effectiveness of prevention and early immunotherapy.

## 1 Introduction

*Helicobacter pylori* is one of the most prevalent chronic infections worldwide and a principal driver of the Correa cascade—from chronic active gastritis to atrophy, intestinal metaplasia, dysplasia, and ultimately gastric cancer (1). Although eradication therapy reduces population-level risk, patients who have already reached atrophy or intestinal metaplasia retain substantial residual risk, underscoring the limits of histology-only stratification and the need for immune-informed frameworks (2). Beyond epithelial mutations and epigenetic drift, mounting evidence shows that dynamic remodeling of the gastric immune microenvironment—oscillating among inflamed, immune-excluded, and immune-desert states—critically shapes progression along this continuum.

In addition to histologic and immune changes, the gastric cancer cascade is accompanied by stepwise molecular and genetic alterations, including *H. pylori* virulence factor-driven signaling, epigenetic drift, and progressive accumulation of oncogenic mutations. Many of these events are closely intertwined with the evolving immune context, shaping antigen presentation, cytokine networks, and the balance between effector and regulatory cells. At disease inception, gastric epithelial and myeloid cells sense microbial patterns via Toll-like receptors (TLRs) and Nucleotide-binding oligomerization domain (NOD)-like receptors, activating the NF-κB/IRF pathways, and inducing IL-1β, IL-6, TNF-α, and CXCL8 that recruit neutrophils and monocytes (3). With chronicity, IL-6/STAT3 and TGF-β/SMAD axes remain engaged, Th1/Th17 polarization intensifies alongside inducible regulatory T cells, and myeloid programs skew toward suppression [tumor-associated macrophages (TAMs) and myeloid-derived suppressor cells (MDSCs)] (4). Antigen-presentation pathways are perturbed—including aberrant epithelial HLA class II—while interferon-γ drives early checkpoint induction such as PD-L1 on epithelial and myeloid compartments, foreshadowing T-cell dysfunction (5). *H. pylori* virulence further rewires immunity: CagA reprograms epithelial signaling and polarity, whereas VacA dampens T-cell proliferation and impairs dendritic maturation, creating high antigenic stimulation but blunted effector function (6, 7).

Contemporary technologies now enable high-resolution profiling of these transitions. Single-cell and spatial transcriptomics, multiplex immunohistochemistry/fluorescence, imaging mass cytometry, and complementary fluid assays delineate cell states, pathway activity, and spatial relationships within intact tissue architecture (8). In parallel, clinical experience with PD-1/PD-L1 inhibitors has highlighted the relevance of pre-existing T-cell inflammation, checkpoint expression, myeloid suppression, TGF-β-rich stroma, vascular abnormalities, and antigen-presentation integrity in gastrointestinal cancers, including gastric cancer (9).

To integrate these strands, we proposed phase-specific “immune snapshots” that concurrently profile three dimensions: cellular composition and activation, pathway activity (IFN-γ/STAT1, IL-6/STAT3, TGF-β/SMAD, NF-κB, WNT/β-catenin, antigen presentation, immune checkpoints, and VEGF/vasculature), and spatial distribution (intralesional, perilesional, and stromal) (4, 10). This framework tracks immune trajectories across *H. pylori*-driven gastric tumorigenesis—encompassing chronic gastritis, atrophy with intestinal metaplasia, and dysplasia/early cancer—and links each phase to actionable assays and interventions, such as stromal normalization prior to immunotherapy or antigenicity priming before checkpoint blockade. Our goal is to complement conventional histopathology with dynamic, mechanism-oriented metrics that delineate windows for intervention.

**Figure 1:**
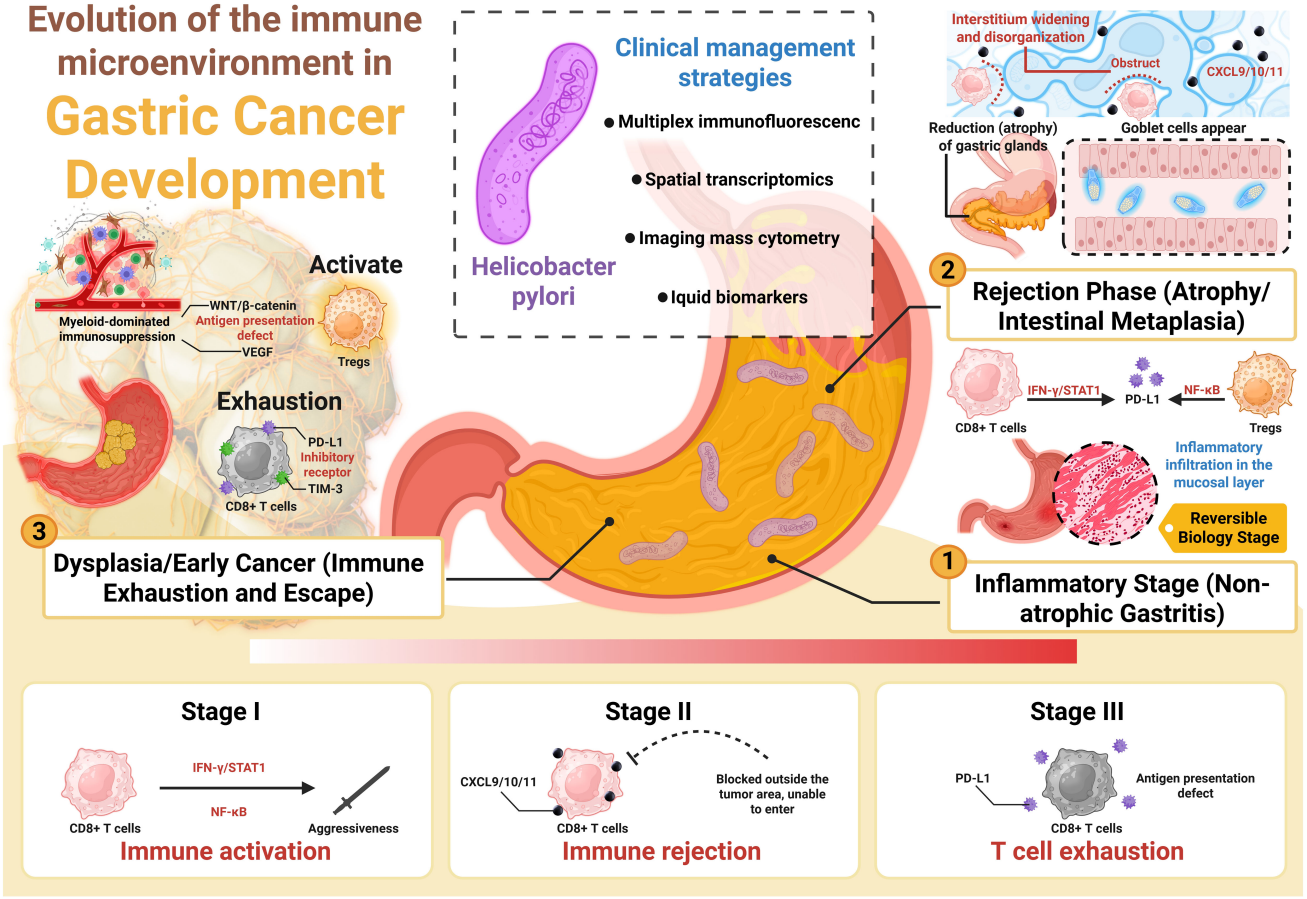
Phase-specific immune snapshots in *Helicobacter pylori*-driven gastric tumorigenesis. Diagram linking three readout layers—who is present, how circuits are engaged, and where they reside—to stage-specific decisions across chronic gastritis, atrophy with intestinal metaplasia, and dysplasia/early cancer, highlighting eradication, stromal/vascular normalization, antigenicity priming, and checkpoint blockade.

The concept of immune contexture highlights the prognostic value of immune-cell type, density, and location, forming the basis of the Immunoscore. Our immune snapshot builds on this by adding functional pathway readouts and barrier features such as CAF/CXCL12 fences, extracellular matrix (ECM) structure, and vascular dysfunction, now measurable by multiplex and spatial assays. In short, contexture addresses who is present and where, while an immune snapshot also asks what they are doing and why access fails or succeeds. Immune snapshots can be aligned with tumor immune microenvironment (TIME) categories. Stage I lesions resemble an inflamed TIME with emerging PD-L1. Stage II resembles an immune-excluded TIME marked by chemokine-rich but entry-poor profiles and CAF/TGF-β/VEGF activity. Stage III splits into inflamed-exhausted or desert/low-presentation states depending on antigen-presentation integrity (11). This mapping clarifies transitions between TIME classes and suggests mechanism-guided interventions. **Figure 1** summarizes the phase-specific immune snapshot framework, aligning who–how–where readouts with stage-specific intervention windows.

## 2 Stage I—chronic non-atrophic gastritis: inflammatory set-point and immune orientation

At the outset of chronic non-atrophic gastritis, the stomach does not jump directly to immune exclusion. It first establishes an inflamed set-point. Epithelial cells and lamina propria myeloid cells sense *H. pylori* through TLR and NOD receptors, activate the NF-κB and IRF pathways, and release IL-1β, IL-6, TNF-α, and CXCL8, which recruit neutrophils and monocytes (12). A low trickle of IFN-γ appears as Th1 priming begins. This reinforces antimicrobial tone and remodels antigen presentation (13). With ongoing exposure, neutrophils remain abundant, and monocyte-derived macrophages and dendritic cells accumulate in superficial glands and the lamina propria. Antigen-processing machinery increases, and aberrant epithelial HLA class II may emerge, prolonging local T-cell stimulation and driving tissue injury (14). The adaptive response tilts toward Th1 and Th17, while inducible Tregs expand as counter-regulation. IFN-γ improves presentation yet also induces PD-L1 on epithelial and myeloid cells, creating one of the earliest checkpoint brakes in *H. pylori* gastritis (15). Virulence factors tune these programs. CagA perturbs epithelial signaling through SHP-2–ERK and weakens junctional integrity, amplifying inflammatory and growth cues. VacA reduces T-cell proliferation and blunts dendritic cell maturation, lowering effector quality despite persistent antigenic stimulation (16). For example, *H. pylori* CagA and VacA not only activate the epithelial signaling pathways (such as SHP-2/ERK and disruption of cell polarity) but also dampen dendritic cell function and T-cell activation, thereby promoting persistent inflammation and early immune evasion. Even before a CAF-rich stroma develops, chronic injury has frayed tight junctions and the mucus layer, increased leakage of microbial patterns, and repeatedly re-engaged TLR and NOD circuits. Early myofibroblast activation, light extracellular matrix deposition, and uneven perfusion together pre-condition the tissue for later immune exclusion.

A pragmatic phase-specific immune snapshot can be obtained without exhaustive testing. Map CD8^+^, Th17, and Tregs across superficial glands and the lamina propria using multiplex immunohistochemistry (IHC) or immunofluorescence (IF) or imaging mass cytometry, and when needed, pair these with single-cell or spatial transcriptomics to couple cellular state with position (17). Add pathway readouts such as NF-κB targets exemplified by CXCL8, p-STAT1 as an IFN-γ response marker, and antigen-processing genes TAP1 and PSMB measured by RNA- In situ hybridization (ISH) or RNA-seq and phospho-IHC (18). Complement these with checkpoint and presentation metrics such as PD-L1 H-score in epithelial and myeloid compartments and epithelial HLA class II aberrancy assessed by multiplex IHC or IF or by flow cytometry on dissociated biopsies. Non-invasive adjuncts from gastric juice or serum, including IL-6 and CXCL8, together with exploratory exosomal PD-L1, can mirror mucosal activation and restraint.

Clinically, this is the reversible window. Eradication remains the principal disease-modifying step. Coupling routine histology with a minimalist immune snapshot that includes the CD8 to Treg ratio, PD-L1 H-score, p-STAT1, and gastric juice IL-6 and CXCL8 can identify patients drifting toward patterns that later consolidate into exclusionary CAF and TGF-β-rich phenotypes (19). This information can guide surveillance and the timing of microenvironment-normalizing strategies.

## 3 Stage II—atrophic gastritis and intestinal metaplasia

Atrophy with intestinal metaplasia marks a decisive bend in the *H. pylori* cascade. Chronic epithelial injury now couples to stromal remodeling and sustained checkpoint engagement, and the mucosa shifts from a chiefly inflamed state toward regulatory and immune-excluded phenotypes (20). Under this pressure, epithelial plasticity, myeloid reprogramming, and barrier dysfunction evolve in step with persistent IL-6 and STAT3 activity together with TGF-β and SMAD signaling. These programs redefine where and when effector lymphocytes can act (21). In clinical practice, this phase often coincides with field-wide epigenetic change and altered acid dynamics that reshape host–microbe interactions and help lock in exclusion circuits even when bacterial burden declines (22). The result is a chemokine-rich microenvironment that becomes progressively less permissive to productive T-cell engagement (23).

Several drivers push tissue toward atrophy and metaplasia. Ongoing *H. pylori* colonization maintains epithelial pattern-recognition signaling and oxidative stress, while CagA and VacA amplify junctional disruption and antigenic drive that bias lineage programs toward atrophy and reprogramming (24, 25). Parietal-cell loss reduces acid and reshapes the physicochemical niche, which favors overgrowth of oral and intestinal taxa and the accumulation of nitrosative by-products that reinforce epithelial injury and metaplastic commitment. In parallel, chronic IL-6 with STAT3 and TGF-β with SMAD link repair programs to immunoregulatory outputs, dampening cytotoxicity and strengthening stromal crosstalk (26). Eradication often lowers inflammation, yet IL-6 with STAT3 and TGF-β with SMAD may remain partially imprinted, sustaining metaplastic fields that no longer depend on pathogen status.

Beyond the morphologic changes, atrophic gastritis with intestinal metaplasia is characterized by a progressive increase in CpG island methylation (“methylation index”) and promoter hypermethylation of key genes such as CDH1, CDKN2A (p16), and RUNX3. These epigenetic lesions arise in the setting of chronic *H. pylori*-driven inflammation and DNA methyltransferase activation and are paralleled by dysregulation of microRNAs that sustain IL-6/STAT3, NF-κB, and TGF-β signaling. Together, these changes link epithelial epigenetic drift to the expansion of immunoregulatory circuits and the establishment of an immunosuppressive microenvironment at this stage.

The immune-cell landscape also tilts. Neutrophils wane relative to monocyte-derived macrophages and dendritic cells, which cluster around metaplastic glands and adopt mixed inflammatory and regulatory states with IL-10 and TGF-β output (14). CD8^+^ and Th1 or Th17 effectors collect at lesion margins, while inducible Tregs and checkpoint-positive myeloid cells rise within and around metaplastic epithelium, raising the threshold for productive cytotoxicity. Epithelial and myeloid PD-L1 increase early, often together with VISTA or TIGIT, and this pattern foreshadows later exhaustion despite interferon priming (27). At the same time, cDC1 frequency and maturation fall relative to cDC2, cross-priming weakens, and CD103^+^ tissue-resident T cells remain constrained by TGF-β and nutrient competition (28).

A practical Stage II immune snapshot is feasible and does not require exhaustive testing. First, quantify CAF density marked by α-SMA, the perilesional ring of CD8^+^ T cells, and the number and location of tertiary lymphoid structures. Map immature and mature dendritic cells and checkpoint-positive macrophages with multiplex IHC or IF and imaging mass cytometry, and add spatial transcriptomics when positioning is needed (18, 29). Next, read out the key pathways. Measure p-STAT3 and p-SMAD2 or p-SMAD3 in epithelium and stroma, and align CXCL12 and CXCL9 to CXCL11 transcripts to cell positions using RNA-ISH or RNA-seq with phospho-IHC (30). Add checkpoint and presentation metrics that capture the chemokine-high yet entry-low paradox, including PD-L1 H-score in epithelial and myeloid cells; patterns of VISTA, TIGIT, and LAG-3; and the status of HLA and TAP1 modules. Finally, document stromal and vascular features that explain failed CD8^+^ accrual, including collagen signatures, microvascular metrics, and hypoxia markers, and consider fluid adjuncts such as serum or gastric juice IL-6 and TGF-β together with exploratory exosomal PD-L1 and CXCL12 for longitudinal monitoring (31–33).

From a clinical standpoint, the dominant picture is immune exclusion. Perilesional CD8^+^ accumulation, high CAF and TGF-β with CXCL12, and engaged checkpoints argue that barrier relief should precede or accompany PD-1 or PD-L1 therapy. Anti-TGF-β, anti-VEGF, and selected matrix-targeted strategies are rational entry points, and surveillance intervals may need to be shortened when exclusion signatures coincide with extensive metaplasia (23). To operationalize this stage, we proposed a minimal immune snapshot that aligns core readouts with corresponding actions (**Table 1**).

**Table 1:** Minimal immune snapshot for atrophic gastritis/intestinal metaplasia.

Dimension	Minimal readouts	Clinical action
T-cell balance and checkpoints	CD8, FOXP3 to derive CD8:Treg ratio, PD-L1 H-score by multiplex IHC/IF or IMC with epithelial vs. myeloid partitioning	Low CD8:Treg with high PD-L1 → close surveillance, consider microenvironment normalization before PD-1/PD-L1
Antigen presentation	HLA class I/II, β2M, TAP1/PSMB by IHC or targeted RNA-ISH	Presentation-low → brief priming to boost antigenicity, then checkpoint blockade
Stroma and CAFs	α-SMA^+^ CAF density and layout, collagen pattern, CXCL12 by ISH	Exclusion-dominant → stromal or vascular normalization, then PD-1/PD-L1
Vasculature and endothelium	Vascular tortuosity, hypoxia markers, optional ICAM-1/VCAM-1	Inflow barrier → endothelial activation or vascular remodeling to enhance T-cell entry
Dendritic cells and TLS	cDC1/cDC2 maturation, TLS number, and location	Weak priming → DC support or priming measures before checkpoint therapy
Fluid monitoring (if tissue limited)	Gastric juice or serum IL-6, CXCL8, exploratory vesicle-associated PD-L1	Longitudinal tracking to guide endoscopy intervals and treatment timing

IHC, immunohistochemistry; IF, immunofluorescence; IMC, imaging mass cytometry; DC, dendritic cell.

## 4 Stage III—dysplasia and early gastric cancer: consolidation of immune escape

As metaplasia progresses to dysplasia and early cancer, the immune terrain hardens into escape. CD8^+^ T cells co-express PD-1, TIM-3, LAG-3, and TIGIT, with lower GZMB and IFN-γ and with poor metabolic flexibility, which together signal entrenched exhaustion (34). Clonotypes narrow and proliferation lags, so interferon-response modules uncouple from effective killing. TOX and NR4A programs and DNA methylation marks consolidate these states, while mitochondrial dysfunction and lipid accumulation limit recovery even when PD-1 is blocked (35, 36).

Myeloid suppression comes to the fore. Tumor-associated macrophages that are CD163^+^ and ARG1^+^, and myeloid-derived suppressor cells expand in dysplastic and early carcinoma niches and release IL-10, TGF-β, and PGE_2_, which blunt T-cell activation and antigen presentation (37). Crosstalk between myeloid cells and cancer-associated fibroblasts sustains suppressive polarization and builds an exclusionary stromal layout. Myeloid aggregates cap gland entrances, and fibroblast cords align with vessels, channeling traffic away from epithelial cores (38).

Tumor-intrinsic escape tightens. HLA class I/II fall or are lost, β2-microglobulin can be altered, and TAP1 and PSMB components are impaired, so neoantigens are less visible despite inflammation. Loss of heterozygosity at HLA together with β2M defects often appears with promoter silencing across antigen-processing genes, creating bottlenecks at several nodes (39). Two subsets stand out as partial exceptions. Epstein–Barr virus (EBV)-positive and Microsatellite instability (MSI)-high disease can retain immunogenicity and respond to PD-1 blockade. Exclusion circuits run in parallel (40). WNT and β-catenin activities drive dendritic cell scarcity and weak priming, while VEGF skews vessels and hypoxia deepens, restricting leukocyte trafficking and reinforcing suppression (41, 42).

A Stage III snapshot can be built with a focused panel rather than an exhaustive one. First, map exhausted CD8^+^ cells defined by the co-expression of PD-1, TIM-3, LAG-3, and TIGIT, together with the abundance and location of tumor-associated macrophages, myeloid-derived suppressor cells, and cancer-associated fibroblasts (43). Chart intratumoral and peritumoral distribution with multiplex IHC or IF, imaging mass cytometry, and spatial transcriptomics. Next, align pathways with position. Read exhaustion signatures, IL-10 with TGF-β with PGE_2_ programs, and WNT with β-catenin and VEGF modules (33). Add presentation and checkpoint metrics to capture concurrent antigen-presentation loss and checkpoint dominance. Track HLA class I/II, β2M, and TAP1 or PSMB9 together with PD-L1 H-scores. Where tissue sampling is limited, add fluid surrogates. Cytokine panels and exosomal PD-L1 can complement tissue readouts, and circulating tumor DNA (ctDNA) together with T-cell receptor (TCR) or B-cell receptor (BCR) fragments can assist longitudinal tracking (44). In addition to tissue analysis, ctDNA could be explored using both mutation-based and methylation-based markers. Candidate mutations include recurrent gastric cancer driver alterations, while promoter methylation of genes such as CDH1, CDKN2A (p16), and RUNX3 may serve as epigenetic readouts of field cancerization. These ctDNA strategies are currently exploratory but may help to non-invasively monitor genetic and epigenetic escape in advanced stages.

Therapeutically, single-agent PD-1 or PD-L1 is unlikely to be durable without circuit relief. Practical triage follows the dominant barrier (43). An inflamed yet exhausted pattern points to PD-1 combined with TIGIT or LAG-3 plus metabolic support. An excluded pattern calls for anti-TGF-β or anti-VEGF or selected matrix-targeted approaches first, followed by PD-1. A low-presentation pattern benefits from a brief priming step, such as radiation or chemotherapy, or oncolytic vectors, before checkpoint therapy. Pair tissue snapshots with fluid surrogates to time barrier relief or priming cycles and to adjust surveillance between interventions (42).

## 5 Discussion

This mini-review proposes phase-specific “immune snapshots” that integrate cellular composition and activation, key pathway activity, and spatial distribution across chronic gastritis, atrophy with intestinal metaplasia, and dysplasia or early cancer. The framework links conventional histopathology to actionable mechanistic readouts and aims to identify reversible immune barriers and optimal windows for intervention.

The conceptual advance is the coupling of immune contexture with functional states, including IFN-γ/STAT1, IL-6/STAT3, TGF-β/SMAD, antigen presentation, and checkpoint pathways, together with CAF programs, extracellular matrix and vascular features, and spatial logic of infiltration. In early lesions, preserved antigen presentation can coexist with PD-L1, indicating a brake that may be lifted by timely therapy. With progression, CAF- and TGF-β-associated exclusion, endothelial non-responsiveness, and downregulated antigen presentation consolidate T-cell exhaustion, which helps explain the limited activity of single-agent PD-1 or PD-L1 therapy.

Clinical translation can rely on concise, standardized assays. Multiplex IHC or imaging mass cytometry can enumerate CD8, Treg, TAM, CAF, and Tertiary lymphoid structure (TLS) while preserving compartmental information. RNA-ISH or targeted RNA sequencing can map p-STAT1, p-STAT3, p-SMAD2/3, and chemokine gradients, combined with HLA, β2M, TAP, and PSMB, to assess antigen processing and presentation. When tissue is scarce, gastric juice or serum measures of IL-6, TGF-β, and vesicle-associated PD-L1 and CXCL12 may serve as longitudinal surrogates. Snapshot-guided decisions include stromal and vascular normalization before immunotherapy, and antigenicity priming and dendritic cell activation before checkpoint blockade. The framework also supports individualized risk stratification and surveillance. In the setting of intestinal metaplasia, high Cancer-associated fibroblast (CAF) density, strong TGF-β/SMAD signaling, perilesional CD8 rings, and vascular abnormalities argue for shortened endoscopic intervals. When the inflammatory set-point is restored, IL-6/STAT3 activity is reduced, and PD-L1 is modest, standard intervals may be appropriate.

Limitations include uncertainty from cross-sectional sampling, anatomic heterogeneity within the stomach, non-equivalence among checkpoint and stromal axes, and the unproven tissue specificity of fluid biomarkers. Future work should prospectively validate snapshot–risk associations in eradication and surveillance cohorts, test barrier-first and priming-then-checkpoint sequences, and establish a minimal standardized panel with operational thresholds for routine care. These sequencing strategies are mechanistically derived and warrant prospective validation with spatial pharmacodynamic readouts to confirm benefit.

## 6 Conclusion

In sum, immune snapshots recast *H. pylori*-associated gastric tumorigenesis as a sequence of modifiable immune states. By converting cellular, pathway, and spatial information into executable assays and sequencing rules, this approach has the potential to improve stratification and therapeutic efficacy beyond histopathology alone.
